# Logging-data-driven lithology identification of conglomerate reservoir by the assistance of integrated machine learning methods

**DOI:** 10.1038/s41598-025-27640-3

**Published:** 2025-11-23

**Authors:** Jiming Liu, Dongjin Xu

**Affiliations:** 1 Key Laboratory of Exploration Technologies for Oil and Gas Resources (Yangtze University), Ministry of Education, Wuhan, 430100 People’s Republic of China; 2 School of Petroleum Engineering, Yangtze University, Wuhan, 430100 People’s Republic of China

**Keywords:** Lithology identification, Machine learning, Logging data driven, Conglomerate reservoir, Energy science and technology, Solid Earth sciences

## Abstract

Lithology is a key parameter in reservoir fine description and evaluation. It is difficult to directly identify reservoir lithology using a single logging curve or conventional cross-plot method due to the mud-gravel mixing in complex reservoirs. The accurate identification of conglomerate reservoir lithology has always been a high-profile issue in reservoir characterization. In this study, over 70 m of cores were observed in detail. And the manually identified lithology after depth correction is matched with five log curves, including GR (natural gamma ray log), DT (Delta-T Compressional log), RHOB (density log), TNPH (thermal neutron porosity log), and M2R1 (shallow high resolution array induced resistivity log). With the logging data as input, three machine learning models were built separately, and the prediction results were compared through different methods, including accuracy analysis parameters and ROC curves. The results show that the machine learning model based on logging data has excellent performance in the lithology prediction of conglomerate reservoir, and the XGBoost model shows the best prediction results with the highest prediction accuracy of 0.902. In addition, the optimal model is interpreted by SHAP method. On the whole, TNPH curve plays the most important role in lithology prediction. This study offers valuable insights into lithology prediction for complex reservoirs.

## Introduction

The accurate lithology identification is a basic and significant issue in reservoir research of petroleum industry^1–4^. Three types of data can be used for lithology identification in different reservoirs: rock cuttings obtained during drilling, cores extracted from the stratum, and logging data. Cuttings logging is considered as a conventional method to obtain lithologies. However, the lithology interval in the well profile is about 1 m and the depth information is commonly inexact. And this method has a certain lag in the drilling process, so it is difficult to accurately reflect the fine change of lithology. Thin section microscopy, a technique that involves observing rock cores or thin sections, can provide accurate lithology identification by allowing geologists to analyze the composition and structure of rocks. However, this method needs amount of cost during the core collection and thin sections preparation. Well logs can provide a lot of information about formation rocks, so the logging curves are commonly used to make lithology identification^5,6^. Conventional cutoff values of logging data can reflect the change of lithology in the vertical profile of a single well. Thus, the logging-data-based lithology identification is usually based on typical log curves that can reflect lithology changes, such as gamma (GR) curve, spontaneous potential curve, well diameter curve, etc., and then the change of shale content in sandstone reservoirs is judged manually or automatically to identify the lithology. Using the cutoff value, spider grams or cross plots, the lithology could be distinguished in conventional reservoirs which has low-GR sandstones characteristic. However, the accurate identification based on logging curve shows difficulties in fan-delta conglomerate reservoir. It is not feasible to use conventional cross-plot or cutoff value to determine the lithology in fan-delta sandstone reservoirs, because if the gravel is acidic igneous rock in the sandstone, its radioactive element content is high, which will lead to high value of the gamma logging curve, and it is easy to be interpreted as mudstone. In addition, the argillaceous component is a near-source deposit, and if its transport distance is close to the reservoir, then the surface of the argillaceous component fails to absorb a large number of radioactive materials, and the natural gamma ray is abnormally low, which shows the characteristic of conventional sandstones. And in some typical reservoirs, gravel and argillaceous components are mixed near the source origin, resulting in higher gamma ray value than sandstone.

Based on well logs, machine learning methods have been widely used for lithology prediction of different types of reservoirs in recent years^7–11^. Leveraging the GR value and its seven derivatives, a comparative study of seven supervised machine learning algorithms was conducted for lithofacies prediction. The stochastic deep forest method emerged as the most effective, achieving a Mean Absolute Error (MAE) of 0.25. Using seven logging curves, including GR and AC, as inputs, a single integrated model and composition of integration models were established to predict eight lithologies and compared in Daniudi gas field of Ordes Basin. Using 76,500 images from tray image as input, ResNeXt-50 model performed best in lithology identification with an accuracy value of 93%^12^. In general, unsupervised and semi-supervised algorithms, supervised algorithms, and deep learning algorithms are used for lithology prediction^13^. However, few lithology identifications in conglomerate reservoirs have been reported, especially fan delta reservoirs which show frequent vertical lithology changes and uneven gravel distribution.

Using raw data from the fan delta conglomerate reservoir, including over 70 m of core data and well logging data, three machine learning models were developed to identify the formation lithology in this study. Accurate lithology identification will help the subsequent fine reservoir description and efficient development.

## Methodology

### Models using boosting algorithms based on regression tree

Boosting, an ensemble learning technique, constructs robust models by incrementally adding weak learners, such as decision trees. This iterative process continuously refines the model by fitting residuals, resulting in high prediction accuracy and robustness. For instance, LightGBM, a member of the Boosting family, offers advantages such as faster training speed, lower memory usage, and improved accuracy over traditional methods like XGBoost. However, the traditional gradient lifting algorithm has the problem of slow training speed and large memory consumption when dealing with large-scale data sets. Boosting can be randomly surmised slightly higher prediction accuracy than weak learning enhancement study for high prediction precision of apparatus, which provides effective new ideas and new methods for machine learning methods^14–17^. Three types boosting algorithms were adopted to identify lithology in this study, including Adaptive Boosting, Light Gradient Boosting Machine, and Extreme Gradient Boosting.

#### Adaptive boosting (Adaboost)

Adaptive Boosting (AdaBoost) regression is a machine learning technique that employs the boosting principle to iteratively combine multiple weak predictors into a robust ensemble model. During training, the algorithm dynamically adjusts the weights assigned to base learners, prioritizing those with smaller prediction errors while diminishing the contribution of high-error models. Through successive iterations, this adaptive weighting mechanism optimizes the ensemble’s performance, ultimately converging to a highly accurate predictor^3,18,19^.

#### Light gradient boosting machine (LightGBM)

The decision trees construction process of LightGBM is similar to the traditional decision tree algorithm, but there are some special features. It uses gradient-based historical information to build a decision tree and selects the optimal split point by calculating the information gain (or similar metric) of each feature. During the construction process, LightGBM also considers factors such as the sparsity of features and unbalance of data to further improve the performance of the model^1,20^.

#### Extreme gradient boosting (XGBoost)

XGBoost (eXtreme Gradient Boosting) is a scalable, distributed gradient-boosting framework built on classification and regression tree (CART) principles, extending traditional decision tree methodologies^21–27^. By integrating base learners such as “GBTree” (CART-based regression trees) and “GBLinear” (linear regressors), it constructs a strong ensemble learner with high predictive accuracy and computational efficiency. Unlike conventional Gradient Boosting Decision Trees (GBDT), XGBoost enhances model robustness through “regularization terms” in its loss function, effectively mitigating overfitting and controlling complexity. A key innovation of XGBoost lies in its optimization strategy: it approximates the objective function using a “second-order Taylor expansion”, incorporating both first and second derivatives to accelerate convergence and improve precision. This approach enables flexibility in defining custom loss functions, provided they are “twice continuously differentiable”. While the framework supports user-defined objectives, it most frequently employs standard loss functions such as mean squared error (MSE) for regression and logistic loss for classification tasks^28–31^.

### Verification methods for classification issues

#### Confusion matrix and accuracy verification parameters

**Fig. 1:**
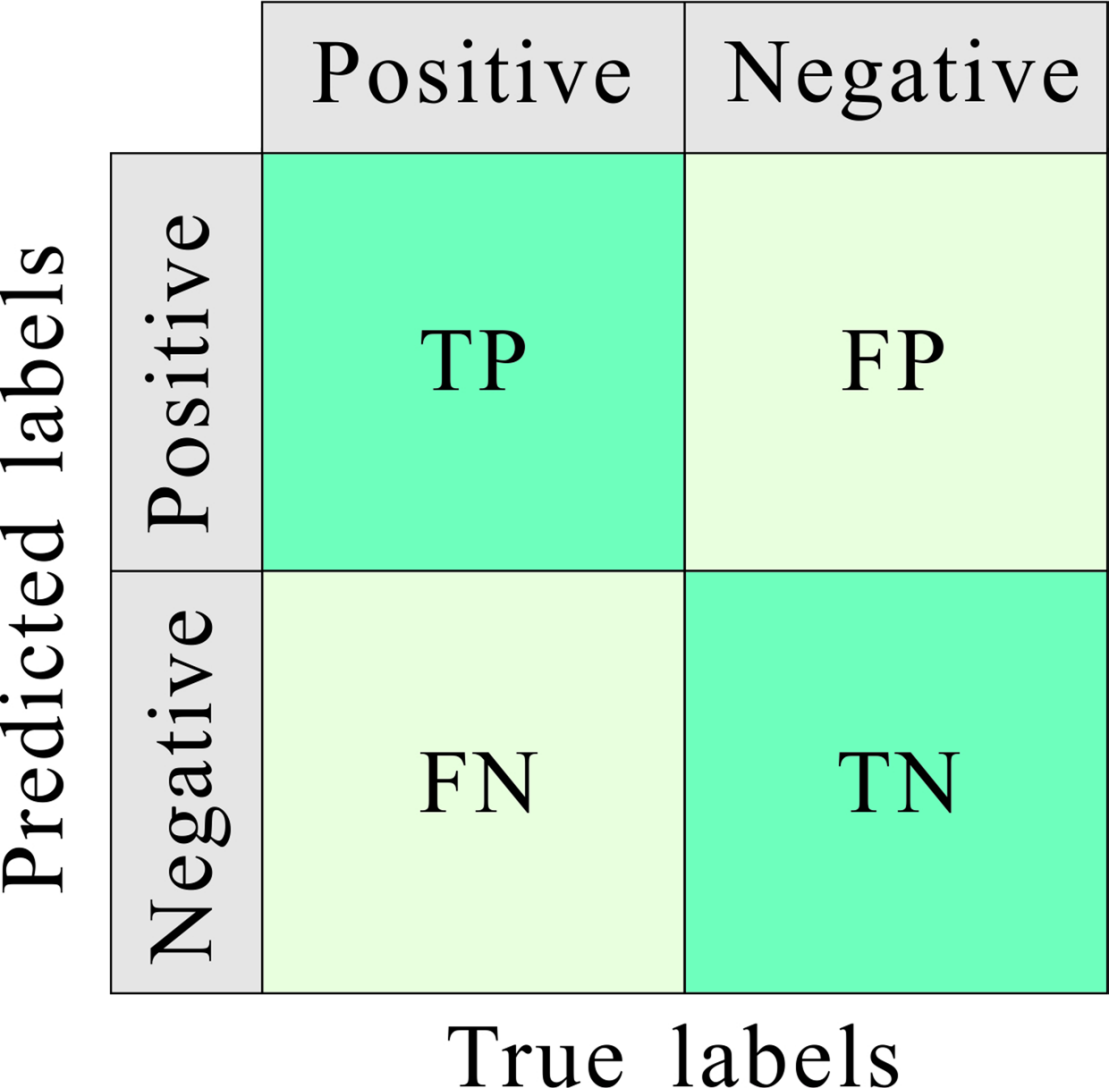
The classical confusion matrix for binary classification issues. TP, true positives; TN, true negatives; FP, false positives; FN, false negatives.

The confusion matrix is a widely employed visualization tool for assessing the performance of classification models, especially in multi-class scenarios. It provides a detailed breakdown of predictions by comparing the model’s outputs against the true labels. And by organizing errors and correct predictions per class, the confusion matrix offers a granular view of model behavior, enabling targeted improvements beyond aggregate metrics like overall accuracy. From the confusion matrix, parameters of model evaluation could be obtained, including precision, recall, F1-score, and accuracy^32–34^. And the classical confusion matrix was shown in Fig. 1.

Precision quantifies the ratio of correctly predicted positive instances to all instances classified as positive. It emphasizes the reliability of positive predictions. And high precision indicates minimal false positives, making it critical in scenarios where FP costs are high.

$$Precision=\frac{TP}{TP + FP}$$

Recall measures the proportion of correctly identified positive instances relative to all actual positive instances. It evaluates the model’s ability to detect relevant cases:

$$Recall =\frac{TP}{TP + FN}$$

And high recall is essential when minimizing false negatives is paramount.

The F1-score harmonizes precision and recall via their harmonic mean. It is particularly useful for imbalanced datasets where optimizing one metric alone may degrade the other. And this metric provides a balanced assessment of classifier performance. The method of F1-score calculation is as follows:

$$\text{F}1-\text{score }=2\times \frac{Precision\times Recall }{Precision+ Recall}$$

And accuracy represents the overall proportion of correct predictions (both positive and negative):

$$\text{Accuracy }=\frac{TP+TN }{TP+TN+FP+FN}$$

#### Receiver operating characteristic (ROC) curve

**Fig. 2:**
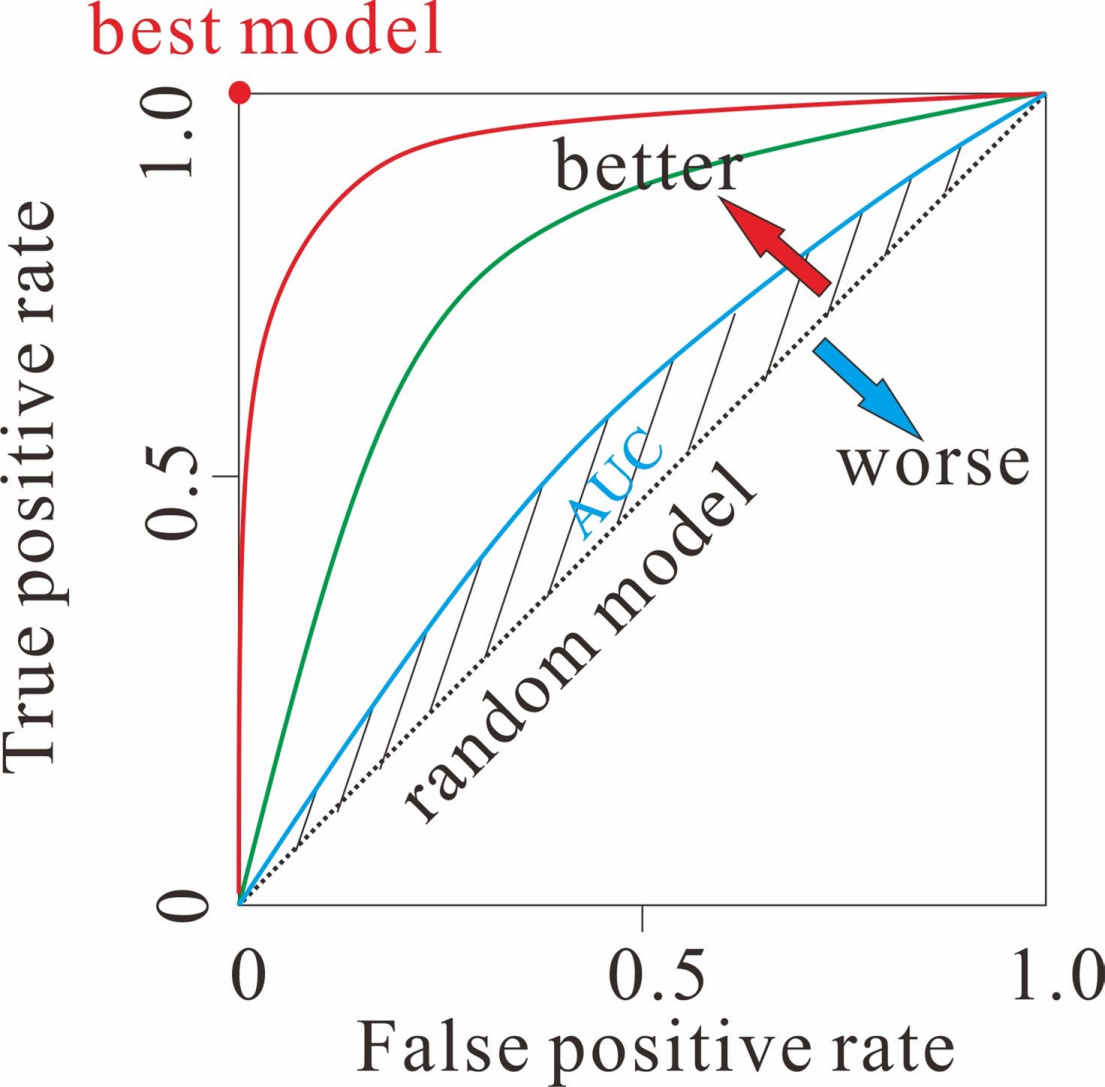
the classical schematic diagram of the ROC curve. Red curve is the characteristic of model 1st; green curve indicates the model 2nd; and the blue one reflects the performance of model 3rd. AUC refers to the area under the curve of model, filled by shadow.

The Receiver Operating Characteristic (ROC) curve, a widely used evaluation tool in binary classification, can be extended to multiclass problems through adaptation strategies. It visualizes the trade-off between the “True Positive Rate (TPR) and False Positive Rate (FPR) across classification thresholds. The ROC curve uses the size of the area under the curve (AUC) to evaluate the model, which was shown in Fig. 2. The Area Under the Curve serves as a key metric for model performance, where a value closer to 1.0 indicates superior diagnostic or predictive ability. Conventionally, an AUC between 0.5 and 0.7 is considered to represent low accuracy, a value between 0.7 and 0.9 suggests moderate accuracy, and an AUC above 0.9 denotes high accuracy. An AUC of 0.5 indicates that the model fails to discriminate between classes and possesses no diagnostic value.

### Shapley additive explanations

Machine learning models are black boxes for users. Due to the difficulties to visualize the prediction process of these models, the interpretability of models remains a challenge and even an issue in the process of using models. In this manuscript, SHAP (Shapley Additive Explanations) method was used to interpreted the best model. SHAP is an interpretative framework derived from cooperative game theory principles, designed to elucidate prediction outcomes across various machine learning models^35,36^. This method quantifies the contribution of individual features through SHAP values—distinct numerical measures assigned to each input variable within a given data sample. It operates by calculating the marginal contribution of each feature to a model’s prediction for a given instance. The result is a set of SHAP values that assign a specific, quantitative importance score to every feature, thereby explaining the model’s output. Theoretically, this approach establishes an additive explanatory model where the collective contribution of all features faithfully represents the final prediction. Theoretically grounded in Shapley value calculations from game theory, SHAP establishes an additive explanation model where features are treated as collaborative participants in the prediction process. This approach ensures mathematically consistent attribution of feature importance while maintaining local interpretability for individual predictions and global insights into model behavior.

## Workflow and data processing

**Fig. 3:**
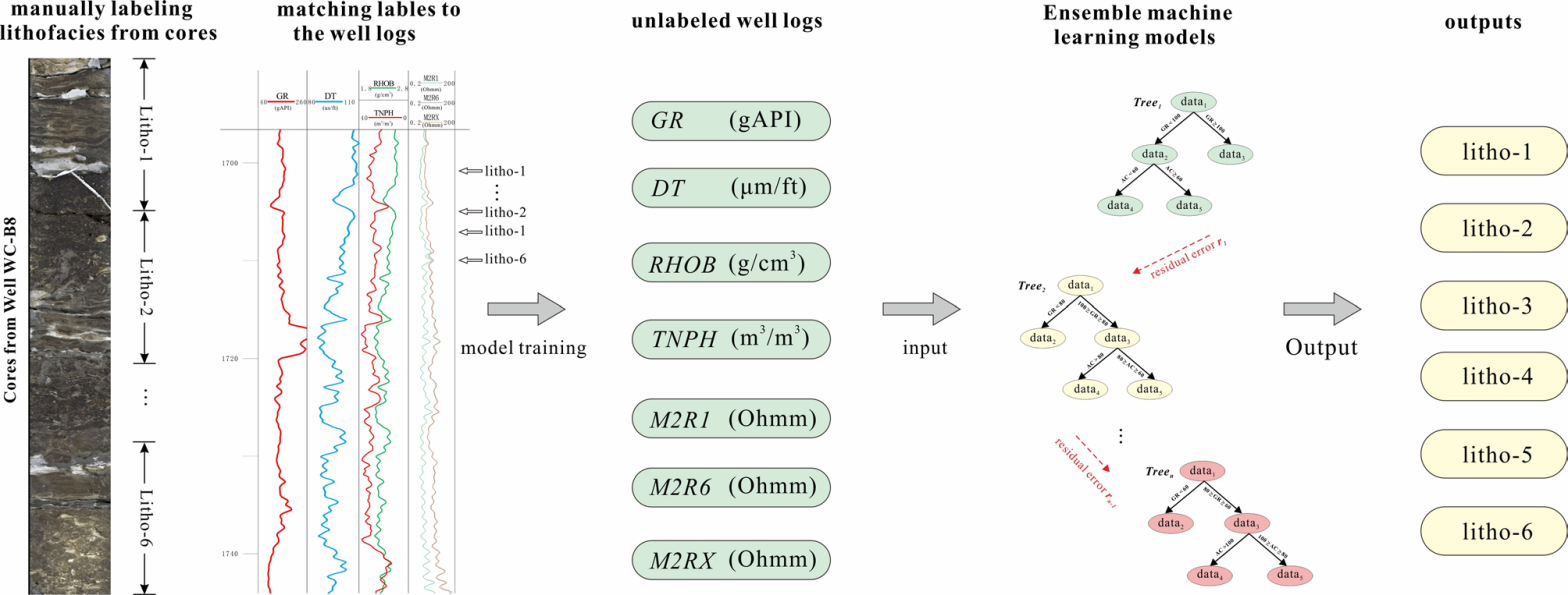
Workflow of logging-data-driven lithofacies identification by the assistance of ensemble machine learning models.

The complete process of lithology identification in conglomerate reservoir based on logging data was established in this study, and the specific steps were show in Fig. 3, including lithology identification of cores, depth matching of the cores, logging data pre-processing, models’ construction and lithology prediction. The entire process is initiated with the lithology identification of cores, which serves as the foundational and most authoritative step, cause the machine learning needs quite correct lithology labels as learning objects. In this step, detailed descriptions of core samples from key wells are conducted, matching different lithologies to measured depth. Subsequently, the depth matching between cores and logging data is performed with rigorous precision. Then the input data were selected and processed. After the dataset was established, the machine learning models of ensemble learning were trained and compared. Finally, the best model was selected for lithology prediction.

### Lithology identification of cores

**Fig. 4:**
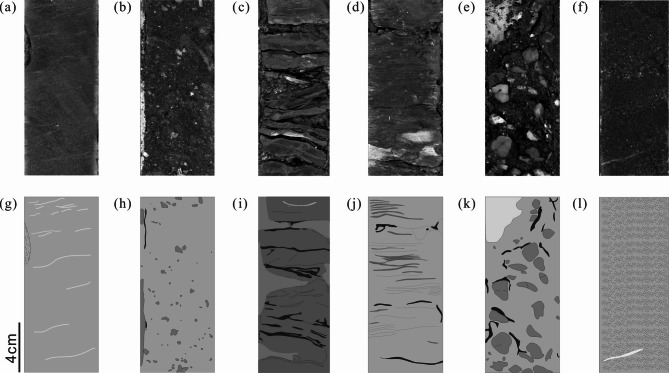
Real photographs and drawings of manually identified lithologies. (**a**) medium-fine sandstone with several muddy bandings; (**b**) pebbly sandstone with small size (less than 8mm) of argillaceous and metamorphic gravels; (**c**) gray mudstone; (**d**) argillaceous sandstone with several sandy bandings; (**e**) conglomerate sandstones with the gravel size over 3.5 cm; (**f**) coarse sandstone with few muddy bandings; (**g**) schematic core drawing of (**a**), highlighting muddy bandings; (**h**) schematic core drawing of (**b**), highlighting gravels; (**i**) schematic core drawing of (**c**); (**j**) schematic core drawing of (**d**), highlighting sandy bandings; (**k**) schematic core drawing of (**e**), highlighting gravels; (**l**) schematic core drawing of (**f**).

The study area is located in the South China Sea, and the reservoir belongs to fan delta front deposits. A total of 71.4 m cores from the formation were observed in detail and six types of lithologies were classified manually, including medium-fine sandstone (litho-1), pebbly sandstone (litho-2), mudstone (litho-3), argillaceous sandstone (litho-4), conglomerate sandstones (litho-5), and coarse sandstone (litho-6). And the real core photographs and drawings are presented in Fig. 4.

### The depth matching of the cores to well logs

**Fig. 5:**
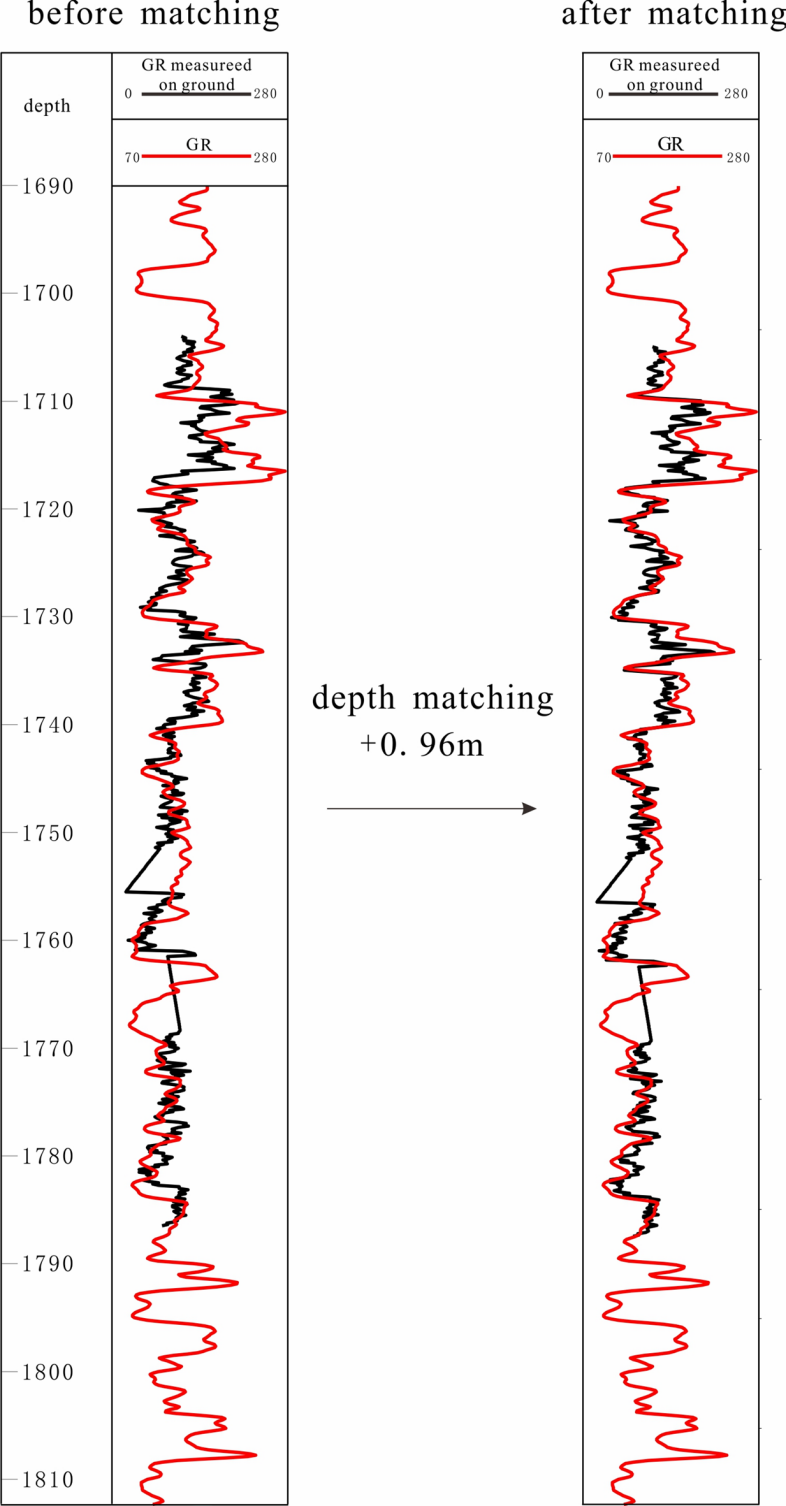
Core depth correction based on GR curves obtained in the well profile and laboratory.

The core observation is done in the laboratory, and the logging curves were derived from the well profile, and the error in depth takes difficulties in the data set construction. Thus, it is significant to make the depth of manual lithology labels and logging curves unified. The depth of collected cores commonly does not match the depth of logging curves because of the measurement error. The measurement error could be obtained through the comparison of GR data obtained using a handheld gamma detector on the ground and the GR in well profile. And the depth of cores should be added 0.96 m to match logging curves, which was shown in Fig. 5. Finally, the lithology labels were matched to the well logging curves.

### Logging data selection and pre-processing

#### Logging data selection

**Fig. 6:**
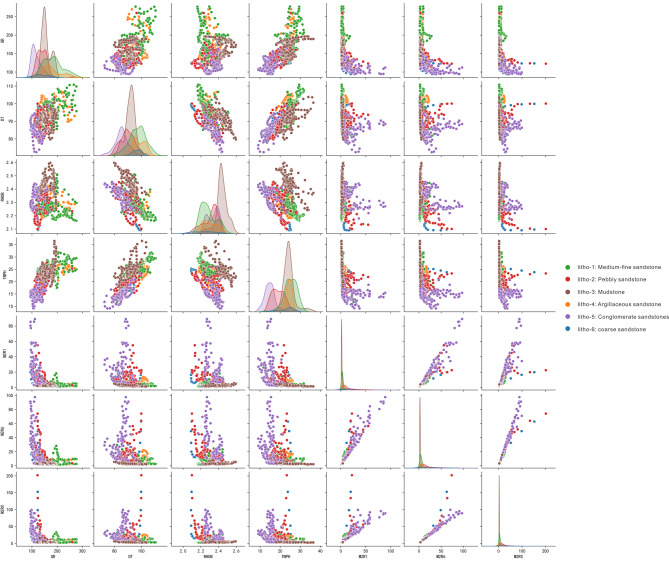
Cross plot of seven logging curves under different lithologies.

From the geological point of view, the GR curve could show response to argillaceous content, and the curves reflecting porosity and flow of rocks can also show the differences among lithologies, including GR (natural gamma ray, gAPI), DT (DTCO, Delta-T Compressional, μm/ft), RHOB (density log, g/cm^3^), TNPH (thermal neutron porosity log, %), M2R1 (shallow high resolution array induced resistivity, ohmm), M2R6 (middle high resolution array induced resistivity, ohmm), and M2RX (deep high resolution array induced resistivity, ohmm). Thus, it’s reasonable to use these seven well logs. The cross plot of seven logging curves under different lithologies was shown in Fig. 6, which showed the degree of differentiation of lithology by intersection of different logging curves. Unfortunately, it is difficult to distinguish the complex six lithologies accurately with two-dimensional logs, but the log’s response to the lithology can be observed.

**Fig. 7:**
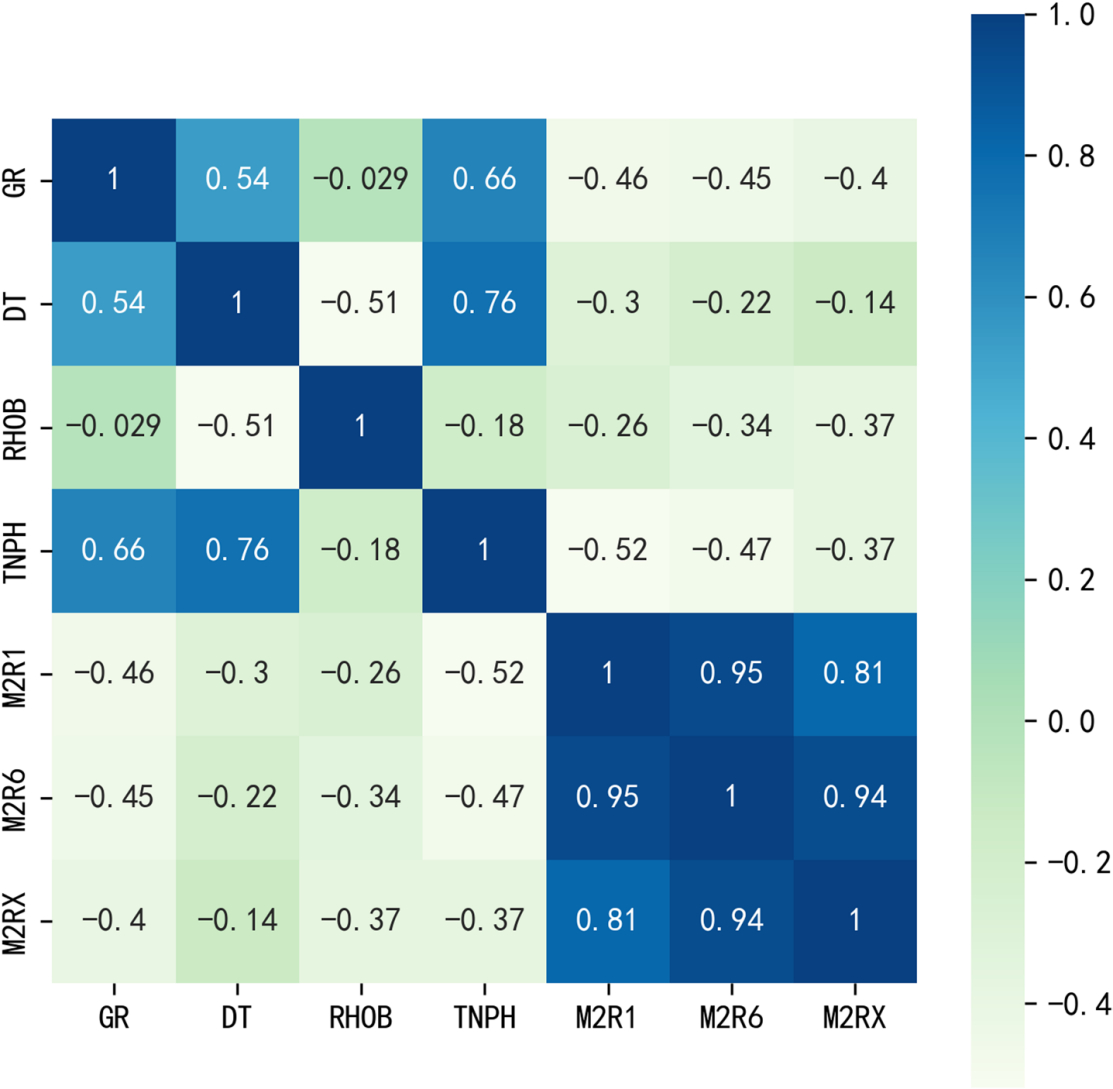
The correlation coefficient heat map of seven logging curves, including GR, DT, RHOB, TNPH, M2R1, M2R6, and M2RX.

However, in the regression or classification machine learning issues, too many parameters will result in higher dimensions of input data, bringing difficulties on model interpretation. Besides, when there exist two or more variables sharing high correlations, the regression model will meet greater risk of over-fitting. Thus, the logging series should be further selected in order to decrease the risk of over-fitting and guarantee the better pattern visualization. The cross-correlation matrix plot of seven well-logging parameters was shown in Fig. 7. In the seven variables, M2R1, M2R6, and M2RX all show the resistivity characteristic of the formation. In addition, the correlation coefficient among them is over 0.8, which brings challenge of over-fitting. High correlation among input features, known as multicollinearity, increases the risk of overfitting because it makes the model’s coefficient estimates highly unstable and sensitive to minor variations in the training data. The model may assign excessive importance to subtle noise within these redundant features, learning patterns that do not generalize. Consequently, while performance on the training data can seem excellent, the model’s ability to perform reliably on new, unseen data is often compromised, leading to high generalization error. Thus, finally selected logging curves were five series, including GR, DT, RHOB, TNPH, and M2R1.

#### Dataset construction and splitting

Combining the manual lithology labels and selected five well longs, the full dataset was constructed according to the depth. And the first ten pieces of data were shown in Table 1.

**Table 1:** The presentation table of the complete data set (the first ten pieces of data).

GR (gAPI)	DT (μs/ft)	RHOB (g/cm^3^)	TNPH (%)		M2R1 (ohmm)	Lithology types
159.658	98.926	2.24	27.117		3.909	Coarse sandstone
161.666	98.939	2.252	26.151		3.662	Coarse sandstone
165.914	99.317	2.254	25.367		3.355	Coarse sandstone
168.514	100.107	2.244	25.027		2.954	Coarse sandstone
169.893	100.846	2.234	25.308		2.664	Coarse sandstone
94.263	90.021	2.279	16.223		34	Coarse sandstone
97.412	91.767	2.291	16.294		18.836	Coarse sandstone
102.83	93.271	2.31	16.637		9.211	Coarse sandstone
115.427	94.207	2.354	17.449		6.559	Coarse sandstone
135.153	94.542	2.398	18.781		4.34	Coarse sandstone

A total of 761 samples were divided into training data and testing data randomly using the “train_test_split” method of PYTHON. And 80% of the full data, 608 samples in total, were used as the training data consisted of x_train and y_train, and the other 20% of the dataset, 153 samples in total, were set as testing data composed by x_test and y_test. The manually labeled lithologies were used as the objective parameter in the whole process. And the five well logs were used as input in the machine learning models. The characteristics of input, both of x_train and x_test were summarized in Tables 2 and 3.

**Table 2:** Statistical summary of 608 input training data. Count, total number of samples; mean, mean value; std, standard deviation; min, minimum value; 25%, quantile at 25%; 50%, quantile at 50%; 75%, quantile at 75%; max, maximum value.

Features	GR (gAPI)	DT (μs/ft)	RHOB (g/cm^3^)	TNPH (%)	M2R1 (ohmm)
Count	608	608	608	608	608
Mean	156.429	92.512	2.339	22.112	7.542
Std	35.460	6.503	0.099	4.740	10.722
Min	94.246	72.415	2.090	9.113	1.264
25%	132.874	88.493	2.256	18.533	2.047
50%	151.283	92.596	2.355	23.240	3.786
75%	176.351	96.370	2.418	25.105	7.683
Max	276.961	110.773	2.597	36.522	89.057

**Table 3:** Statistical summary of 153 input testing data. Count, total number of samples; mean, mean value; std, standard deviation; min, minimum value; 25%, quantile at 25%; 50%, quantile at 50%; 75%, quantile at 75%; max, maximum value.

Features	GR (gAPI)	DT (μs/ft)	RHOB (g/cm^3^)	TNPH (%)	M2R1 (ohmm)
Count	153	153	153	153	153
Mean	155.383	92.134	2.361	22.159	7.584
Std	33.650	6.679	0.101	4.841	12.376
Min	94.787	77.998	2.096	10.510	1.272
25%	136.513	87.334	2.294	19.473	1.898
50%	149.665	92.612	2.384	23.415	3.372
75%	172.658	95.163	2.426	25.184	7.875
Max	276.589	109.789	2.568	31.531	85.452

## Results and discussion

In this section, the construction processes of three machine learning models are presented using the processed dataset, including Adaboost, LightGBM, and XGBoost models. Specially, the model tuning process and kernel selection are also discussed in this section. Finally, the best models are interpreted using the SHAP method.

### Models construction and hyperparameters tuning

Using the segmented data set, three machine learning models based on boosting method are constructed, including Adaboost, LightGBM, and XGBoost.

The three hyper-parameters of boosting models, named max_depth, learning_rate and n_estimators, play important roles in model performance and robustness. In order to obtain a more effective model, the tuning process of max_depth, learning_rate and n_estimators were discussed based on grid-search method. Grid search is a classical, straightforward, and powerful method for hyperparameter optimization. It works by systematically exhaustively evaluating all possible combinations of hyperparameters within a predefined set and employs cross-validation (e.g., k-fold) to robustly assess the performance of each combination. And for every type of machine learning models, learning_rate, max_depth, and n_estimators have been optimized by operating within a large data range.

Generally, the ideal learning_rate fluctuates between 0.05 and 0.3 for different problems. In this study, the interval between 0.01 and 5 was search by a step of 0.01. For Adaboost model, it obtained best performance when the Learning_rate was set as 0.4. And the Learning_rate of LightGBM was set as 0.7. XGBoost model shows best prediction accuracy with the Learning_rate of 0.1.

Max_depth is also a key hyperparameter in the boosting model, which means the max depth of decision tree in the forest. For the Adaboost model, It does not have a direct max_depth parameter by itself, but it can be used in conjunction with decision trees, and decision tree models have max_depth parameters. In the Scikit-learn library, when using AdaBoostClassifier, setting the decision tree as a weak classifier and specifying its max_depth parameter could meet the need of Max_depth for better performance. For Adaboost and XGBoost models, the Max_depth was set as 8 to make better prediction. And it was set as 10 in the LightGBM model.

In the boosting models, the value of n_estimators is the number of trees in the “forest”. The N_ estimators was set as 80, 40, and 100 in Adaboost, LightGBM and XGBoost, respectively. The hyperparameters search ranges and optimal values were summarized in Table 4.

**Table 4:** Hyperparameters search ranges and optimal values.

Models	Hyperparameters		
	Learning_rate	Max_depth	N_ estimators
Adaboost	[0.01,5], 0.4	[1,50], 8	[10,300], 80
LightGBM	[0.01,5], 0.7	[1,50], 10	[10,300], 40
XGBoost	[0.01,5], 0.1	[1,50], 8	[10,300], 100

### Predicted results of three ensemble models

**Fig. 8:**
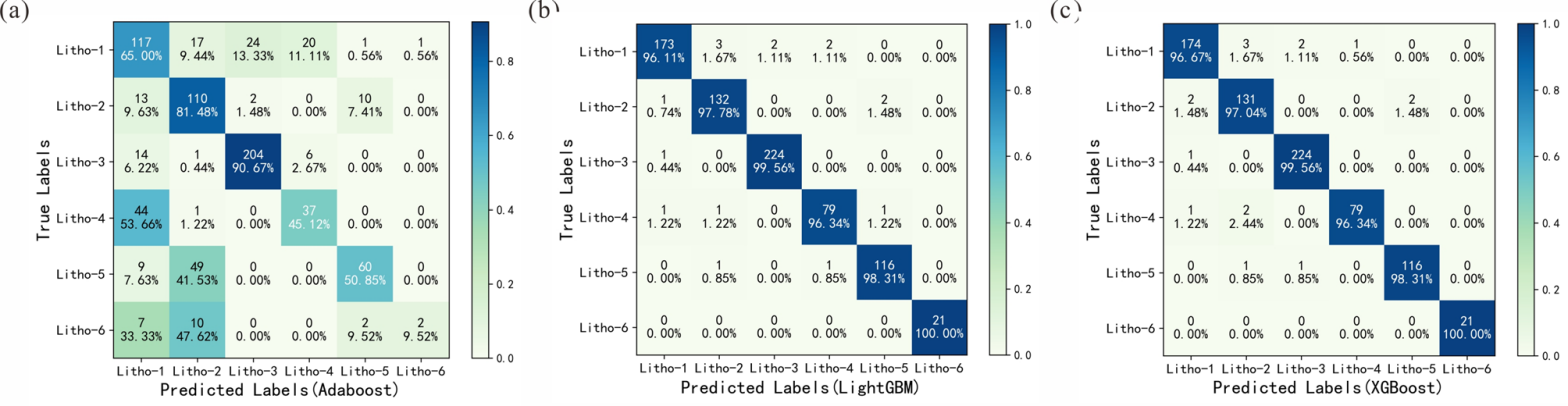
Confusion matrixes showing the performance of different models. (**a**) Adaboost model; (**b**) LightGBM model; (**c**) XGBoost model. litho-1: medium-fine sandstone; litho-2: pebbly sandstone; litho-3: mudstone; litho-4: argillaceous sandstone; litho-5: conglomerate sandstones; litho-6: and coarse sandstone.

Confusion matrixes showed the performance of different models, which were shown in Fig. 8. Compared with LightGBM model and XGBoost model, the Adaboost model showed worse with more incorrect identification results, especially in the argillaceous sandstone and conglomerate sandstones. According to the confusion matrixes, LightGBM and XGBoost models showed same performance on four lithologies, including mudstone, argillaceous sandstone, conglomerate sandstones, and coarse sandstone. And the difference of the two models existed in the medium-fine sandstone and pebbly sandstone prediction. A total of 173 medium-fine sandstone samples were predicted correctly by LightGBM, which was 174 in XGBoost model. And in the pebbly sandstone prediction, LightGBM showed better performance than XGBoost model with one correctly predicted sample.

Using the tuned hyperparameters, the models were constructed and the predicted results of three ensemble models on testing data were compared. The detailed accuracy verification parameters were listed in the Tables 5, 6 and 7, including Adaboost, LightGBM, and XGBoost model.

**Table 5:** Prediction results analysis table of Adaboost model.

Lithologies	Adaboost (testing data)		
	Recall	Precision	F1-score
Medium-fine sandstone	0.590	0.590	0.590
Pebbly sandstone	0.786	0.595	0.677
Mudstone	0.933	0.894	0.913
Argillaceous sandstone	0.529	0.692	0.600
Conglomerate sandstones	0.652	0.882	0.750
Coarse sandstone	0.000	0.000	0.000
Average	0.582	0.609	0.588
Accuracy	0.725

**Table 6:** Prediction results analysis table of LightGBM model.

Lithologies	LightGBM (testing data)		
	Recall	Precision	F1-score
Medium-fine sandstone	0.821	0.914	0.865
Pebbly sandstone	0.893	0.833	0.862
Mudstone	0.978	0.957	0.967
Argillaceous sandstone	0.824	0.824	0.824
Conglomerate sandstones	0.913	0.875	0.894
Coarse sandstone	1.000	0.000	0.000
Average	0.905	0.734	0.735
Accuracy	0.895

**Table 7:** Prediction results analysis table of XGBoost model.

Lithologies	XGBoost (testing data)		
	Recall	Precision	F1-score
Medium-fine sandstone	0.846	0.917	0.880
Pebbly sandstone	0.893	0.806	0.847
Mudstone	0.978	0.936	0.957
Argillaceous sandstone	0.824	0.933	0.875
Conglomerate sandstones	0.913	0.913	0.913
Coarse sandstone	1.000	0.000	0.000
Average	0.909	0.751	0.745
Accuracy	0.902		

For different types of lithology, models showed different performance. The accuracy was the overall proportion of correct predictions, which shows the model’s performance intuitively. The XGBoost model reached the best performance with an accuracy of 0.902. And the accuracy on testing data was 0.725 and 0.895 of Adaboost and the LightGBM models.

XGBoost and LightGBM models always showed better performance in the prediction process than the Adaboost judged by the other verification parameters. For example, XGBoost model showed satisfactory performance on the mudstone prediction, with a recall of 0.978, a precision of 0.936, and a score of 0.957. While the three parameters on mudstone prediction in Adaboost model were 0.933, 0.894, and 0.913, respectively. XGBoost and LightGBM models showed similar performance on lithology, and the verification parameters showed subtle differences. For example, XGBoost model obtained a recall of 0.846 in the medium-fine sandstone prediction, while the recall is 0.821 in the LightGBM model when predicting the medium-fine sandstone. In order to see the comparison more intuitively, the average values of recall, precision, and F1-score for six lithologies were calculated and compared. In the Adaboost mode, the average value of recall for all lithologies was 0.582, and it was 0.905 and 0.909 in LightGBM and XGBoost model. Combining these three synthesis parameters, the XGBoost model outperformed the LightGBM model by a small margin.

**Fig. 9:**
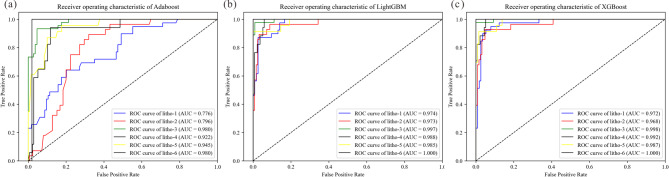
ROC curves showing the performance of different models. (**a**) Adaboost model; (**b**) LightGBM model; (**c**) XGBoost model. litho-1: medium-fine sandstone; litho-2: pebbly sandstone, litho-3: mudstone, litho-4: argillaceous sandstone, litho-5: conglomerate sandstones, litho-6: and coarse sandstone.

Finally, the ROC curves were used to compare the models’ performance quantitatively. The AUC value indicates the degree of prediction for every type of lithology. For example, in the Adaboost model, the AUC value of medium-fine sandstone is 0.776, while it is 0.974 and 0.972 in LightGBM and XGBoost models. It means for the medium-fine sandstone prediction, LightGBM showed the best performance in the three models. As shown in Fig. 9, XGBoost performed best in overall lithology prediction, by comparing AUC values of all lithologies under different models.

### Visualization and interpretation of the best model

#### Predicted lithology comparison on well profile

**Fig. 10:**
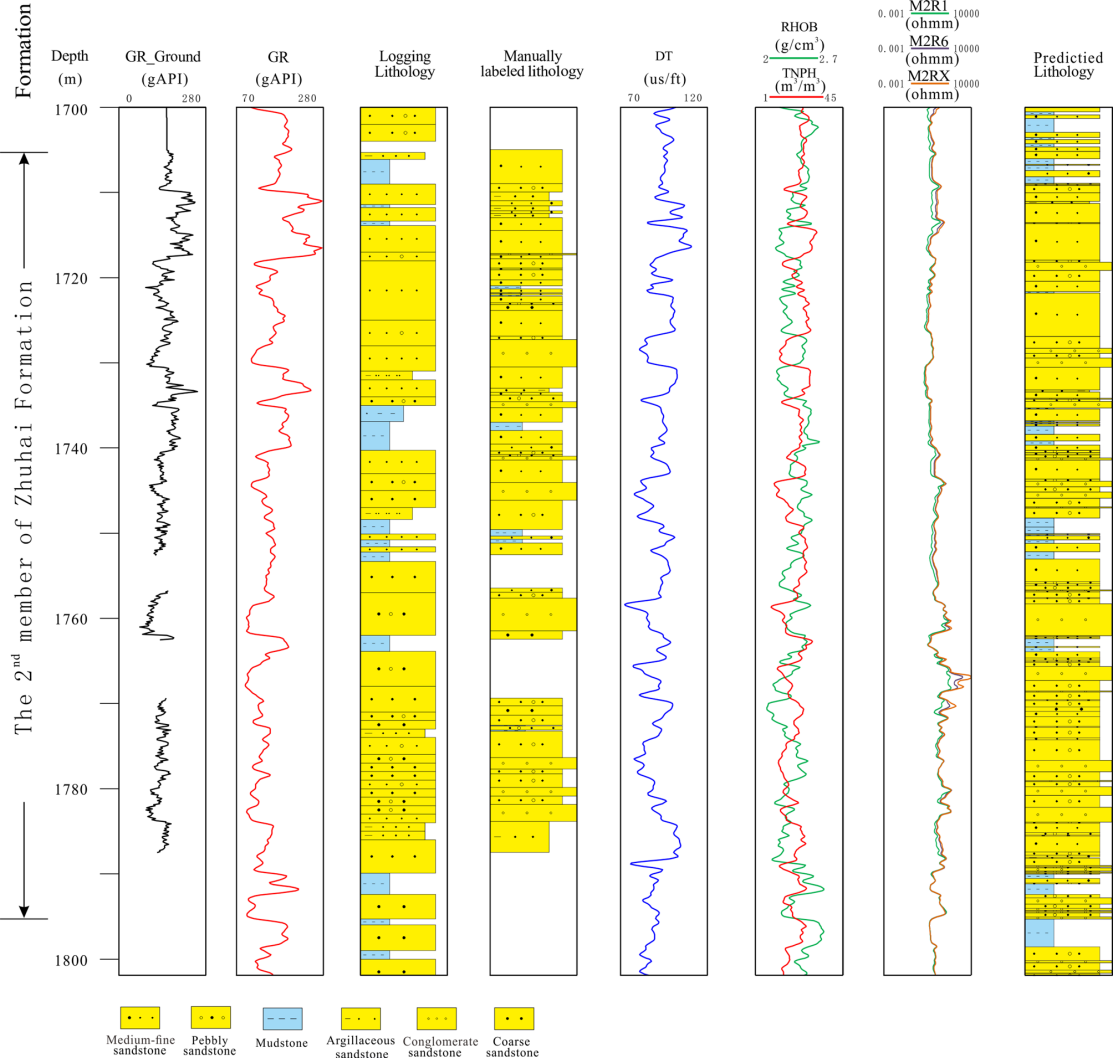
Comprehensive diagram showing the continuous lithology predictions in the well profile based on the trained XGBoost model.

Based on the XGBoost model, continues lithology on the well profile was obtained, and it was compared with logging lithology and manually labeled lithology. It is obvious that the predicted lithology has more accurate and finer lithology than logging lithology. And as shown in Fig. 10, it also indicates the complete lithology profile could be obtained through machine learning models based on well logs.

#### Hyperparameters of the XGBoost model

Visualization of model parameters is necessary for the reproduction of experimental results. Through the order of “get_ parameters” in XGBoost model, the hyperparameters of the best model is visualized as follows: {‘objective’: ‘multi:softprob’, ‘use_label_encoder’: True, ‘base_score’: 0.5, ‘booster’: ‘gbtree’, ‘colsample_bylevel’: 1, ‘colsample_bynode’: 1, ‘colsample_bytree’: 0.8, ‘enable_categorical’: False, ‘gamma’: 0, ‘gpu_id’: -1, ‘importance_type’: None, ‘interaction_constraints’: ‘‘, ‘learning_rate’: 0.1, ‘max_delta_step’: 0, ‘max_depth’: 8, ‘min_child_weight’: 1, ‘missing’: nan, ‘monotone_constraints’: ‘()’, ‘n_estimators’: 100, ‘n_jobs’: 8, ‘num_parallel_tree’: 1, ‘predictor’: ‘auto’, ‘random_state’: 1440, ‘reg_alpha’: 0, ‘reg_lambda’: 1, ‘scale_pos_weight’: None, ‘subsample’: 0.5, ‘tree_method’: ‘exact’, ‘validate_parameters’: 1, ‘verbosity’: None, ‘eval_metric’: [‘logloss’, ‘auc’, ‘error’], ‘nthread’: -1, ‘seed’: 1440}.

#### SHAP values of different variables

**Fig. 11:**
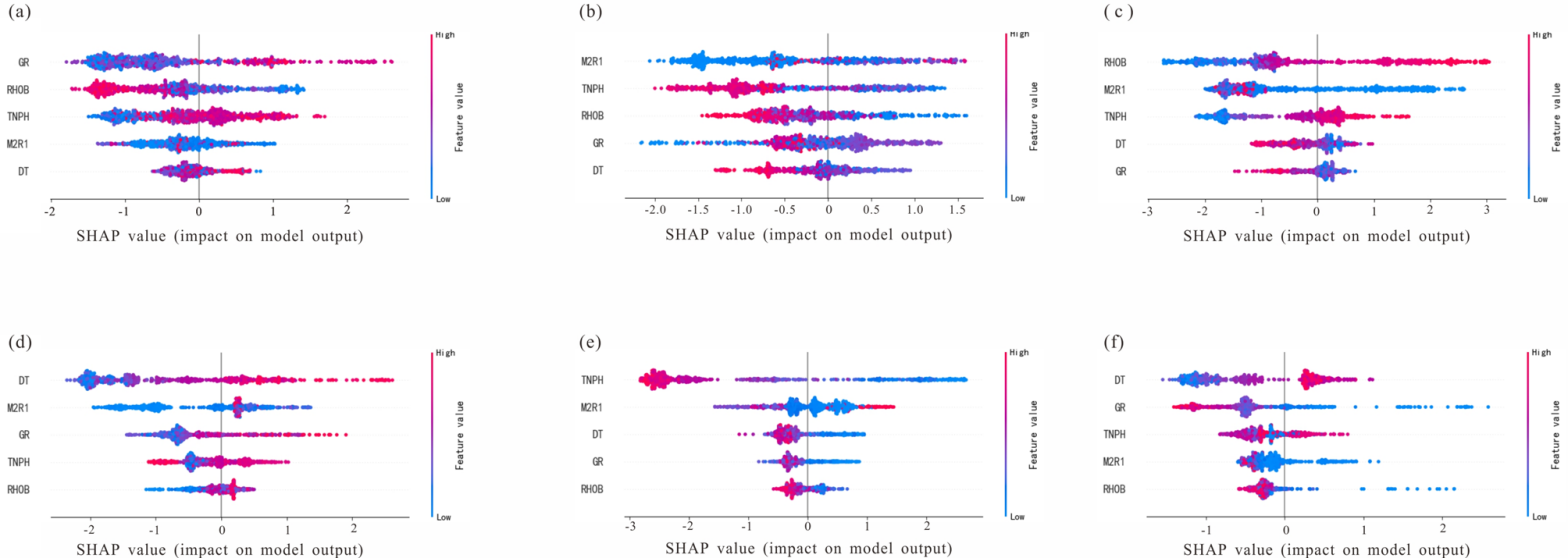
Comprehensive analysis diagram of SHAP values for all variables in different lithologies perdition. SHAP values of all variables in (**a**) medium-fine sandstone perdition; (**b**) pebbly sandstone perdition; (**c**) mudstone perdition; (**d**) argillaceous sandstone perdition; (**e**) conglomerate sandstones perdition; (**f**) coarse sandstone perdition. Blue dots indicate a small variable value and red data points indicate a large variable value.

Different well logs played different roles in the prediction process of different lithologies, which was shown in Fig. 11. For example, the in the Fig. 11a, GR Has the widest range of its SHAP values. It indicated that when the model making the medium-fine sandstone perdition, GR had the greatest probability of having the greatest contribution to the predicted results. While it showed the minimal contribution in mudstone perdition shown in Fig. 11c. And RHOB has the highest contribution in mudstone prediction. Through the interpretation of a particular lithology, the contribution of the well logs to the result could be quantified and the working process of the model could be obtained.

#### Global model interpretation of XGBoost model

After the local interpretation is complete, the global interpretation is crucial to the understanding of the XGBoost model.

**Fig. 12:**
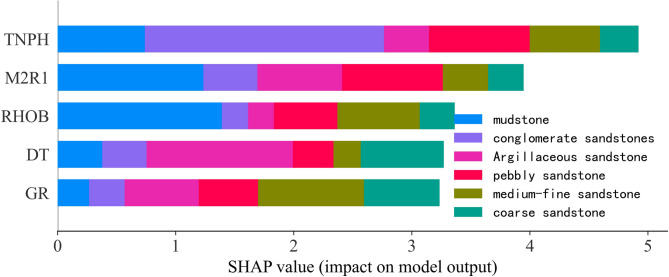
Global interpretation to the XGBoost model. The bars in different colors show the global contribution of a particular variable to a given lithology prediction.

As shown in Fig. 12, the global contribution of different well logs in lithology prediction were interpreted. For example, in the bule bar in Fig. 12, it was obvious that RHOB played the most important role in mudstone prediction, and then there came the well logs of M2R1, TNPH, DT, and GR. This result also verifies that GR is difficult to accurately and intuitively correspond to lithology changes in the lithology prediction of conglomerate reservoirs.

## Conclusion

This study establishes a machine learning-based method for the automated lithology classification of sandstone-conglomerate reservoirs using conventional well logs (GR, DT, RHOB, TNPH, M2R1). Several important conclusions were concluded as follows.

1. A method for automatically identifying the lithology of the conglomerate reservoir is proposed using well logs as inputs, including GR, DT, RHOB, TNPH, and M2R1.
2. Three machine learning models were constructed to make a rapid prediction of lithology using well-logging data, including Adboost model, LightGBM model, and the XGBoost model. The XGBoost showed the best performance among the three models, with an accuracy of 0.902.
3. Based on the interpretation tool of SHAP, the global contribution of different well logs in lithology identification were quantified. For the all lithologies, TNPH played the most important role in lithology prediction, and then there came the well logs of M2R1, RHOB, DT, and GR. And for one specific lithology type, SHAP values for all variables could meet the need for quantitative ranking of contributions.

## Data Availability

The data used in the manuscript have been uploaded on the wibsite of https://figshare.com/authors/jiming_liu/22416130, including well logging series and lithology types. In addition, the codes could be shared if asked by email.
